# Antipsychotic drugs increase Neuregulin1β1 serum levels in first-episode drug-naïve patients and chronic schizophrenia with suggestions for improving the treatment of psychotic symptoms

**DOI:** 10.1186/s12888-022-03856-9

**Published:** 2022-03-25

**Authors:** Haidong Yang, Wen Pan, Wenhuan Xiao, Man Yang, Jianchun Xu, Jin Li, Xiaobin Zhang

**Affiliations:** 1 Department of Psychiatry, The Fourth People’s Hospital of Lianyungang, The Affiliated KangDa College of Nanjing Medical University, Lianyungang, 222003 P.R. China; 2 Institute of Mental Health, Suzhou Psychiatric Hospital, The Affiliated Guangji Hospital of Soochow University, Suzhou, Jiangsu 215137 P.R. China; 3 Department of Psychiatry, Affiliated WuTaiShan Hospital of Medical College of Yangzhou University, Yangzhou, 225003 P.R. China; 4 Department of Clinical Laboratory, The Fourth People’s Hospital of Lianyungang, The Affiliated KangDa College of Nanjing Medical University, Lianyungang, 222003 P.R. China

**Keywords:** Schizophrenia, Neuregulin1, Therapeutic effects, Pathophysiological mechanism

## Abstract

**Background:**

Neuregulin1 (NRG1) plays a role in neuronal migration, regulation of synaptic plasticity, and neural survival, and has been considered to be among the candidate genes for schizophrenia. This study focused on the variations in serum NRG1β1 levels following antipsychotic treatment and the relationship between NRG1β1 levels and improvements in psychotic symptoms among first-episode drug-naïve (FEDN) patients and patients with chronic schizophrenia.

**Methods:**

A total of 100 patients with schizophrenia were recruited and compared with 79 matched healthy controls. All patients had been drug-naïve for at least four weeks. Serum NRG1β1 levels and positive and negative syndrome scale (PANSS) scores were measured at baseline and after four weeks. Serum NRG1β1 levels were measured using sandwich enzyme-linked immunosorbent assays (ELISAs).

**Results:**

Baseline NRG1β1 levels were significantly lower in patients with schizophrenia than in healthy controls. NRG1β1 levels increased significantly following antipsychotic treatment. NRG1β1 levels gradually increased with declining PANSS scores and its three subscales during antipsychotic therapy. The levels of NRG1β1 increased significantly in responders after four weeks of treatment, although nonresponders showed no such effect. Correlation analyses showed that the levels of NRG1β1 were negatively correlated with the duration of illness and positively correlated with improvement in symptoms.

**Conclusion:**

The levels of serum NRG1β1 and the therapeutic effects gradually increased following treatment, indicating that NRG1β1 may be an indicator of therapy, and that it may also be associated with the pathophysiological mechanism causing schizophrenia, although this possible pathway requires further investigation.

**Supplementary Information:**

The online version contains supplementary material available at 10.1186/s12888-022-03856-9.

## Introduction

Schizophrenia is one of the most severe and complex mental disorders and affects nearly 1% of the global population; its rising prevalence has led to a greater burden in underdeveloped countries [1]. Nevertheless, only approximately half of patients with schizophrenia receive satisfactory treatment because its symptoms vary, its underlying pathogenetic mechanisms have yet to be elucidated, and antipsychotic treatments, and their side effects are poorly understood [2]. The evidence suggests that neurotrophic factors (NTFs) play a crucial role in the pathogenesis of schizophrenia, and neurotrophic hypotheses have gained momentum with the development of antipsychotic therapies [3, 4].

Neuregulin (NRG) is an NTF closely related to the development of the central nervous system [5] and comprises NRG1-4 isoforms, among which NRG1 and its receptor ErbB4 are thought to be among the susceptibility genes for schizophrenia. Moreover, alterations to the NRG1 and ErbB4 signaling pathways are involved in neuronal migration, regulation of synaptic plasticity and neural survival, which may be the neurobiological underpinnings of schizophrenia [6, 7]. Most importantly, an increasing number of studies have reported that the NRG1-ErbB4 signaling pathway is related to glutamatergic, GABAergic and dopaminergic neurotransmission [8]; this signaling pathway also plays a vital role in the psychopathological mechanism of schizophrenia, and may be useful in evaluating the effect of antipsychotic medication therapies or new drug developments [9]. Subsequently, evidence from studies of mouse models has demonstrated that overexpression of heterozygous NRG1 type III in mice regulates schizophrenia-relevant behaviors, indicating that NRG1 is associated with abnormal behavioral phenotypes in schizophrenia [10]. In addition, a systematic review has implied that antipsychotic drugs can modify some abnormal behaviors in NRG1 and ErbB4 knockout transgenic mouse models, and can affect the expression of NRG1/ErbB4 and the functioning of NRG1–ErbB4 signaling pathways [11].

A postmortem study reported that NRG1 type I mRNA expression in hippocampal tissue increased in patients with schizophrenia patients compared with controls [12]. In contrast, Parlapani et al. found that the expression of NRG1 type I is decreased and isoform II is increased in the prefrontal cortex of elderly patients with schizophrenia, indicating that the altered expression of NRG1 may play an important role during neurodevelopment, neuronal migration and differentiation in schizophrenia [13]. In addition, an imaging genetic study suggested that the risk T allele of SNP8NRG243177, which has been identified as a functional single-nucleotide polymorphism (SNP) in a regulatory domain of NRG1 that impacts NRG1 function, can contribute to the enlargement of the lateral ventricles observed in early phases of schizophrenia [14]. Genetic variation in NRG1 has also been associated with hippocampal volume reductions in Icelandic patients with schizophrenia and in nonaffected family members [15].

A previous study found that NRG1 mRNA expression in peripheral lymphocytes is abnormally low in first onset schizophrenia, and that this expression gradually increases in antipsychotic treated patients, demonstrating that NRG1 mRNA may serve as a potential therapeutic marker [16]. Another study in an Australian cohort showed that serum NRG1β1 protein levels are lower in clozapine-treated patients with schizophrenia than in healthy controls, while there is no difference in the mRNA expression of NRG1 type III [17]. Research by Kastin indicated that NRG-1 can enter the spinal cord and brain by a saturable receptor-mediated mechanism, indicating the possibility that NRG-1-b1 may be a promising candidate in central nervous system therapeutics [18].

Taken together, these studies have provided evidence that abnormal expression and function of NRG1 may be associated with pathophysiological mechanisms and therapeutic effects in schizophrenia, although these studies have failed to show a consistent relationship between NRG1 and schizophrenia. Accordingly, we hypothesized that antipsychotic treatment may alter NRG1 levels. This study was designed to determine: (1) whether serum NRG1β1 levels were altered after atypical antipsychotic treatment; and (2) whether alterations in serum NRG1β1 levels were correlated with improvements in psychotic symptoms among first-episode drug-naïve (FEDN) patients and patients with chronic schizophrenia. Previously, only a few studies have explored the relationship between serum NRG1β1 levels and the therapeutic effect of antipsychotic treatment in patients with schizophrenia of Han Chinese ancestry.

## Methods

### Subjects, assessment and study procedures

A total of 69 (male/female = 34/35) FEDN patients and 31 (male/female = 21/10) chronic patients with schizophrenia were recruited from Wu Tai Shan Hospital, YangZhou, China. The medication of chronic patients with schizophrenia was stopped for 4 weeks. The total male/female ratio was 55/45, the mean age (± standard deviation SD) was 35.07 ± 11.42 years, the mean period in education was 10.40 ± 3.43 years, the mean age at schizophrenia onset was 29.59 ± 10.64 years, and the mean duration of illness was 5.36 ± 7.58 years. All patients were confirmed to have schizophrenia by using the Structured Clinical Interview of the Diagnostic and Statistical Manual-IV (SCID). Psychotic symptoms were assessed by two experienced psychiatric specialists using positive and negative syndrome scale (PANSS) scores before treatment and four weeks after treatment. The interrater correlation coefficient for the PANSS score was > 0.8. The exclusion criteria were epilepsy, mental retardation, dementia, traumatic or chronic brain injury, alcohol or substance dependence/abuse, thyroid diseases, diabetic peripheral neuropathy, and patients currently undergoing psychopharmacological treatment.

Patients were treated with atypical antipsychotic drugs, including: clozapine (*n* = 7), risperidone (*n* = 16), ziprasidone (*n* = 5), quetiapine (*n* = 8), olanzapine (*n* = 6), amisulpride (*n* = 13), aripiprazole (*n* = 8), or a combination of these antipsychotics (*n* = 37). Antipsychotic dosages were personalized according to the clinical symptoms of each participant. Participants were divided into responder and nonresponder groups according to symptom improvement on the basis of a 25% reduction in the baseline PANSS score after four weeks of treatment [19].

We also recruited seventy-nine controls in good physical health (male/female = 44/35) from the local community in Yangzhou, with an average age of 35.92 ± 11.00 years and a mean time in education of 12.48 ± 3.77 years. The health status of the subjects was identified by physical examination and laboratory tests. Participants who had a family history of mental illness or were diagnosed with any Axis I disorders were excluded.

All participants in this study gave informed written consent, which was approved by the Ethics Committee of Wu Tai Shan Hospital.

### NRG1β1 assessment

We collected venous blood samples from patients at baseline and after four weeks of medication, while healthy controls were only sampled at baseline. All venous blood samples from both patients and healthy controls were collected from the forearm vein between 07:00 and 09:00. The samples were tested by the same investigator and our research team was blinded to the clinical status of this study. Peripheral blood was separated by centrifugation at 3000 g for 15 min and then stored at -80 °C until use. Serum NRG1β1 levels were tested using sandwich enzyme-linked immunosorbent assays (ELISAs) in accordance with the manufacturer’s instructions (DY377; R&D Systems, Minneapolis, MN, USA). The serum NRG1β1 levels were expressed in ng/mL, and the intra-assay and interassay variation coefficients were < 5%, with 0.125 ng/mL as the lower detection limit.

### Statistical analysis

Statistical Package for Social Sciences (SPSS) 19.0 was used to analyze all of the data. Chi-square tests were used to compare categorical variables. The distribution of the data was examined using the Kolmogorov‒Smirnov test. Quantitative variables are expressed as the mean ± standard deviation (SD). Independent sample t-tests or paired samples t-tests or analysis of variance (ANOVA) were performed for normality continuous variables. Analysis of covariance (ANCOVA) was used to analyze potentially confounding variables such as sex, age and years of education. Relationships between serum NRG1β1 levels and normally distributed continuous variables were assessed using Pearson’s correlation analysis, while relationships between serum NRG1β1 levels and nonparametric continuous variables were evaluated using Spearman’s correlation analysis. We used stepwise regression analysis to investigate the associations of NRG1β1 serum levels with demographic data and clinical characteristics. Effect size determination and power calculation were carried out using the G*Power3 program [20]. The results were considered significant when *p* < 0.05.

## Results

### Demographic data

Table 1 shows the detailed data on the demographic differences between patients with schizophrenia and healthy controls. Except for age and years of education, there were no significant differences in sex, smoking or body mass index (BMI) among FEDN patients, patients with chronic schizophrenia and healthy controls (*p* > 0.05). There were no significant correlations between NRG1β1 levels and sex, age or education in the two groups (*p* > 0.05). The mean dose of antipsychotic drugs (chlorpromazine equivalent) was 638.04 ± 268.95 mg/day. In the patient group, duration of illness, age at schizophrenia onset, and antipsychotic dose were not associated with NRG1β1 levels (all *p* > 0.05). Table 2 shows the significant reduction in PANSS scores and its three subscales after treatment (all *P* = 0.000). The results of *P* values of the Kolmogorov‒Smirnov test were presented in the supplementary materials.

**Table 1:** Demographic data of patients with schizophrenia and healthy controls

	FEDN patients (*n* = 69)	Chronic patients with schizophrenia (*n* = 31)	Controls (*n* = 79)	*F/χ*^*2*^*/t*	*P*
Sex (M/F)	34/35	21/10	44/35	2.960^a^	0.228
Age (years)	32.68 ± 11.14	40.39 ± 10.35	35.92 ± 11.01	5.434^b^	0.005
Time in education (years)	10.38 ± 3.59	10.45 ± 3.09	12.48 ± 3.77	7.402^b^	0.001
BMI (kg/m^2^)	23.92 ± 2.77	24.51 ± 2.35	23.35 ± 3.36	1.825^b^	0.164
Smoking	40/29	17/14	35/44	2.932^a^	0.231
Age schizophrenia onset	30.68 ± 10.81	27.16 ± 10.00	-	1.540^c^	0.127
Duration of illness (years)	1.98 ± 3.17	12.90 ± 9.04	-	-6.545^c^	0.000

^a^*χ*^2^ test

^b^ANOVA

^c^independent samples t-test

*FEDN* first-episode drug-naïve, *BMI* body mass index

**Table 2:** Reduction in PANSS scores and its three subscales before and after treatment

	Before treatment	After treatment	*t*	*P*
PANSS total score	77.49 ± 6.22	49.21 ± 11.18	21.459	0.000^a^
P subscore	24.34 ± 7.56	16.13 ± 5.48	8.999	0.000^a^
N subscore	20.84 ± 7.33	13.30 ± 4.21	8.779	0.000^a^
G subscore	32.31 ± 4.42	19.78 ± 6.24	16.535	0.000^a^

^a^paired samples t-test

*PANSS* Positive and Negative Syndrome Scale

### NRG1β1 levels

**Fig. 1:**
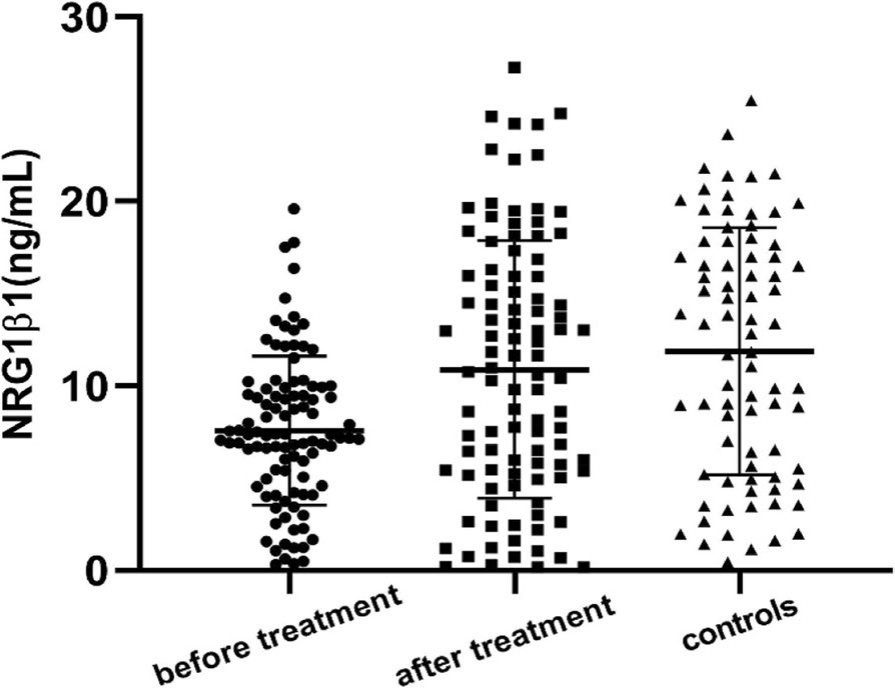
NRG1β1 levels in the patient and control groups before and after antipsychotic drug treatment

Independent sample t-tests showed that baseline serum NRG1β1 levels in the patient group were significantly decreased compared with those in control group (7.58 ± 4.03 vs. 11.87 ± 6.69 ng/mL, F = 42.918, t = -5.030, *P* < 0.000) with an effect size of 0.36 and a power of 0.78. Paired sample t-tests showed that baseline serum NRG1β1 levels were significantly lower than posttreatment levels among patients with schizophrenia (7.58 ± 4.03 vs. 10.89 ± 6.97 ng/mL, t = -4.341, *P* = 0.000), with an effect size of 0.27 and a power of 0.58 (Fig. 1). Significant differences remained when accounting for covariates such as sex, age, education, smoking, BMI, and chlorpromazine equivalent (*P* < 0.000). In addition, no significant differences in NRG1β1 levels were detected between the patient group after antipsychotic treatment and healthy controls (t = 0.951, *P* = 0.343; Fig. 1). In the patient group, there was no significant correlation between the PANSS total and its subscale scores, or NRG1β1 serum levels (all *P* > 0.05). The other correlation analysis data can be found in the supplementary materials.

### Relationship between NRG1β1 and clinical efficacy in responders

**Fig. 2:**
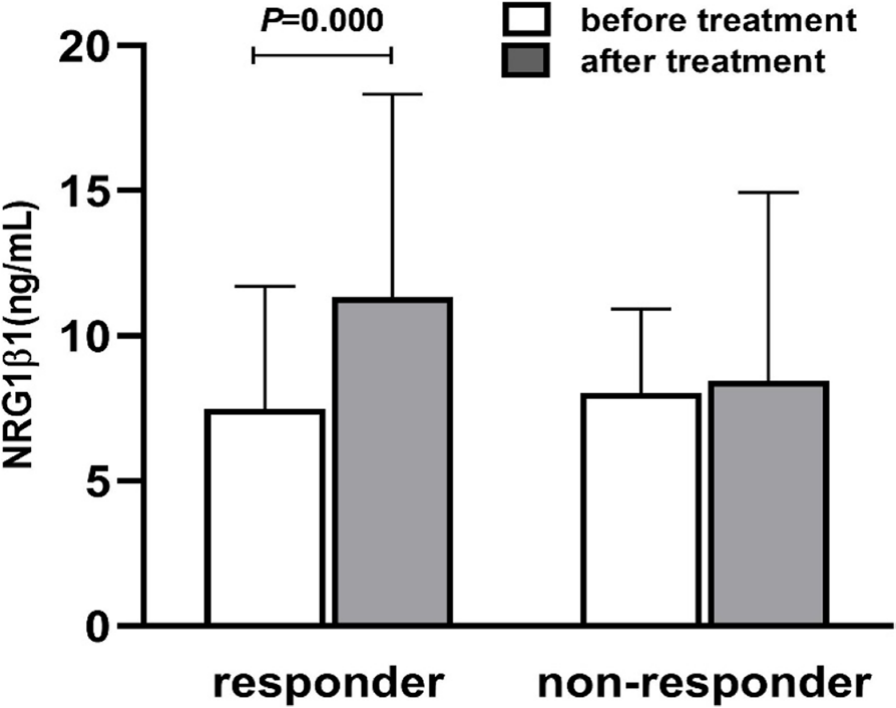
NRG1β1 levels in the responder and nonresponder groups before and after antipsychotic drug treatment

Patients with schizophrenia who had a posttreatment PANSS score greater than 25% different from their baseline score were considered responders. After four weeks of antipsychotic drug treatment, 85% of patients were classified as responders, while 15% of patients were placed into the nonresponder group. In the responder group, serum NRG1β1 levels were markedly higher after treatment (11.32 ± 7.00 ng/mL) than before treatment (7.50 ± 4.21 ng/mL) (t = 4.555, *P* = 0.000, Fig. 2), with an effect size of 0.31 and a power of 0.66. In contrast, there was no significant difference in the level of NRG1β1 before and after treatment (8.03 ± 2.91 vs. 8.44 ± 6.49 ng/mL) in the nonresponder group (t = 0.250, *P* = 0.806, Fig. 2).

In the responder group after treatment, Pearson’s correlation coefficients showed a correlation between NRG1β1 levels and improvements in symptoms (*r* = 0.223, *P* = 0.04). Multiple regression analyses adjusting for potentially confounding variables showed no relationships between NRG1β1 levels and sex, age, smoking, BMI, time in education, age at schizophrenia onset, or chlorpromazine equivalent, but confirmed a relationship between NRG1β1 levels and the duration of illness (beta = -0.234, *P* = 0.032).

## Discussion

The results of this study demonstrated that 1) NRG1β1 levels were lower in FEDN patients with chronic schizophrenia than in healthy controls; 2) levels of NRG1β1 increased significantly after antipsychotic treatment along with improvements in psychotic symptoms; and 3) NRG1β1 levels were negatively associated with the duration of illness in the responder group. To date, few studies have examined the relationship between serum NRG1β1 levels and antipsychotic medication in FEDN patients with chronic schizophrenia.

This study found decreased serum levels of NRG1β1 in FEDN patients and chronic patients with schizophrenia, which is consistent with the results of another study of patients of Han Chinese ancestry, indicating significantly lower NRG1 levels in participants with first episode schizophrenia and chronic schizophrenia than in healthy controls [21]. However, there are contradictory findings reported in the literature; one study reported elevated cleavage of neuregulin-1 by β-secretase (BACE1) in the plasma of patients with schizophrenia and a relationship between BACE1-cleaved-NRG1 activity and both disease severity and disease duration [22]. Moreover, Yamamori et al. measured the mRNA expression levels of NRG1 in immortalized lymphocytes and found no difference in the expression of the NRG1 gene between patients with schizophrenia and controls [23]. Numerous factors may account for these differences, such as demographic differences (age, sex, time in education, body mass index, smoking, or ethnicity), age at schizophrenia onset, drug treatment, duration of illness, disease severity, or methodological or test protocol differences. Different degrees of genetic variations or differences in the subtypes of patients with schizophrenia may also have contributed to the discrepancies in the reported serum NRG1 levels. The decreased serum NRG1β1 levels found in this study are consistent with those observed in the brains of patients with schizophrenia. For example, significantly reduced NRG-1α protein levels have been found in the white matter of the prefrontal cortex [24]. Furthermore, Hashimoto et al. reported a decrease in NRG-1 type II/type I and type II/type III mRNA expression ratios in the prefrontal cortex (PFC) of schizophrenic patients [25]. Nonetheless, our evidence suggests that variation in NRG1β1 levels may be responsible for the pathophysiology and clinical manifestation of schizophrenia, even though the underlying mechanisms remain largely unknown.

Notably, this study demonstrated that elevated NRG1β1 levels are associated with the early-stage therapeutic effects of typical antipsychotic treatments. The detected NRG1β1 levels were markedly higher in responders after four weeks of treatment, whereas no differences were observed in nonresponders. Additionally, in a clinical study of a 2-week treatment of first-onset patients with schizophrenia (who had not taken antipsychotics before) with the antipsychotics risperidone and quetiapine, the NRG1 mRNA expression of peripheral blood lymphocytes (PBLs) gradually and significantly increased after therapy, compared with the pretreatment levels [16]. Although different atypical antipsychotic drugs were administered to responders, we speculate that increased NRG1β1 plays an important part in achieving effective medication and improvement of psychotic symptoms through its effects on the various signaling pathways. The exact pharmacological and pathophysiological mechanisms remain obscure and warrant further investigation.

Several animal studies have shown that NRG1/ErbB4 (the NRG1 receptor) expression can be altered by treatment with drugs for chronic psychosis. Dang et al. found that haloperidol and risperidone both increased the expression of NRG1 in the brains of rats during a 4-week treatment [26]. Hahn et al. reported that NRG1-induced ErB4 activation is significantly reduced in the brains of mice treated with haloperidol compared with controls treated with vehicle alone, and that enhanced NRG1 signaling may contribute to N-methyl-D-aspartate (NMDA) receptor hypofunction in schizophrenia [27]. In particular, one previous study found that NRG1 (both endogenous and exogenous) plays an important role in maintaining evoked GABA release in the rat prefrontal cortex (GABAergic dysfunction is implicated in schizophrenia), suggesting that NRG1 may be involved in interactions with other signaling pathways which could be useful in understanding the pathogenesis of schizophrenia and the effects of treatment [28]. However, there was no significant effect of chronic haloperidol treatment on serum Ig-NRG1 immunoreactivity in monkeys after eight weeks of haloperidol treatment [29]. An in vitro study showed that NRG1 protein expression is upregulated in human fetal brain aggregates exposed to clozapine; however, these effects were not observed in haloperidol exposed brain aggregates [30]. Taken together, these studies support a potential mode of therapeutic action for antipsychotics via NRG1–ErbB4 signaling which contributes to treatment efficacy in controlling schizophrenia symptoms.

Generally, a longer duration of illness hinders treatment response and is related to more severe negative symptoms. Earlier intervention could shorten the duration of illness and lead to a more favorable prognosis in patients with schizophrenia [31]. Our analysis demonstrated that there was a negative correlation between NRG1β1 level and duration of illness in responders. The results showed that patients with longer histories of schizophrenia may have lower NRG1β1 levels, indicating that the length of illness is associated with nervous system impairments, leading to perturbed NRG1β1 synthesis, release and modulation of neuronal activity. Shorter periods of illness inflict less damage on the central nervous system and may allow for better therapeutic outcomes. Kataria et. al. showed that NRG1/ErbB participates in different neurodevelopmental processes and is involved in dopaminergic neurotransmission as well as the survival of dopaminergic neurons [32]. Interestingly, we found a positive correlation between NRG1β1 levels and improvement in symptoms in responders. One explanation for the underlying mechanism is that the relationship between NRG1β1 levels and improvement in symptoms may be the result of the body’s ability to properly establish positive feedback mechanisms to counter neuroimmune system damage. Therefore, in view of the relationship between NRG1β1 levels and the duration of illness and symptoms, we hypothesize that NRG1β1 may be an indicator of therapeutic effect and its progress in schizophrenia through interactions between neurotransmitters and neurotrophic biomarkers. This potential mechanism requires further study.

There are some limitations to this study. First, although the effect size was estimated prior to the study, the sample size was still small. Second, different atypical antipsychotics may have different influences on serum NRG1β1 levels, and further work is needed to compare the effects of different antipsychotics on NRG1β1 levels. Third, the follow-up period was relatively short, and longer follow-up will be needed in the future to provide further evidence for revealing the pathophysiological mechanisms of schizophrenia. Finally, we enrolled chronic patients with schizophrenia, admittedly, age at schizophrenia onset, duration of illness, type of antipsychotic medication and its side effects, number of disease relapses, BMI may have influences on the NRG1β1 levels.

In conclusion, this study demonstrated that serum NRG1β1 levels increased in FEDN patients and patients with chronic schizophrenia after medication with atypical antipsychotic drugs in addition to improvements in psychotic symptoms. These altered NRG1β1 levels may be associated with the progression of illness and response to treatment. This study provides evidence that NRG1β1 may be involved in the pathophysiological and potential therapeutic mechanisms of schizophrenia, although the underlying pathways still require further investigation.

## Supplementary Information

**Additional file 1: Table 1.** *P* values of the Kolmogorov‒Smirnov test in schizophrenia patients and controls. **Table 2.** Correlation analysis of NRG1β1 concentration with general status and clinical symptoms before and after treatment.

## Data Availability

The datasets used and analyzed during the current study are available from the corresponding author on reasonable request.

## Abbreviations

ANCOVA: Analysis of covariance
BMI: Body mass index
ELISA: Enzyme-linked immunosorbent assay
FEDN: First-episode drug-naïve
NMDA: N-methyl-D-aspartate
NRG: Neuregulin
NTF: Neurotrophic factor
PANSS: Positive and Negative Syndrome Scale
PBLs: Peripheral blood lymphocytes
PFC: Prefrontal cortex
SCID: The Structured Clinical Interview of DSM-IV
SD: Standard deviation
SNP: Single-nucleotide polymorphism
SPSS: Statistical Package for the Social Sciences
