# Supplementary material for: Antipsychotic drugs increase Neuregulin1β1 serum levels in first-episode drug-naïve patients and chronic schizophrenia with suggestions for improving the treatment of psychotic symptoms
Source: BMC Psychiatry. 2022 Mar 25;22:217. doi: 10.1186/s12888-022-03856-9 (PMC8957169; doi:10.1186/s12888-022-03856-9)
Supplement: Supplementary file 1 — Additional file 1: Table 1. P values of the Kolmogorov‒Smirnov test in schizophrenia patients and controls. Table 2. Correlation analysis of NRG1β1 concentration with general status and clinical symptoms before and after treatment. [file 12888_2022_3856_MOESM1_ESM.docx]

Table 1: P values of the Kolmogorov‒Smirnov test in schizophrenia patients and controls.

|  | Schizophrenia (n=100) | Controls (n=79) |
| --- | --- | --- |
| Age | 0.156 | 0.200 |
| Time in education | 0.068 | 0.200 |
| BMI | 0.112 | 0.057 |
| Age at schizophrenia onset | 0.132 | - |
| Duration of illness(years) | 0.054 | - |
| Baseline NRG1β1 | 0.136 | 0.102 |
| After treatment NRG1β1 | 0.082 | - |
| PANSS total score | 0.168 | - |
| P subscore | 0.053 | - |
| N subscore | 0.200 | - |
| G subscore | 0.123 | - |
| Chlorpromazine equivalent |  |  |

Table 2 Correlation analysis of NRG1β1 concentration with general status and clinical symptoms before and after treatment.

|  | Baseline NRG1β1 | | After treatment NRG1β1 | |
| --- | --- | --- | --- | --- |
|  | *r* | *P* | *r* | *P* |
| Age | 0.000 | 0.998 | 0.108 | 0.284 |
| Time in education | -0.118 | 0.242 | -0.171 | 0.090 |
| BMI | 0.066 | 0.511 | 0.090 | 0.422 |
| Age at schizophrenia onset | -0.001 | 0.995 | 0.192 | 0.055 |
| Duration of illness(years) | 0.034 | 0.737 | -0.131 | 0.194 |
| PANSS total score | -0.013 | 0.897 | 0.105 | 0.297 |
| P subscore | -0.023 | 0.823 | 0.062 | 0.538 |
| N subscore | 0.001 | 0.990 | -0.038 | 0.709 |
| G subscore | 0.018 | 0.857 | 0.104 | 0.302 |
| Chlorpromazine equivalent | -0.001 | 0.990 | 0.023 | 0.817 |
